# From Biomass Waste to Multifunctional Biochar: Tailored Preparation and Emerging Applications in Energy, Environment, and Sensing

**DOI:** 10.3390/molecules31162893

**Published:** 2026-08-19

**Authors:** Xi Luo, Yiheng Lu, Guangteng Bai, Zaiyong Jiang, Xianglin Zhu

**Affiliations:** 1 Institute for Energy Research, School of Chemistry and Chemical Engineering, Jiangsu University, Zhenjiang 212013, China; 2 School of Chemistry & Chemical Engineering and Environmental Engineering, Weifang University, Weifang 261061, China

**Keywords:** biochar, photocatalytic, fuel cell, pollution remediation, sensing

## Abstract

Biochar is a porous carbonaceous material synthesized through the pyrolysis of diverse biomass resources, including agricultural and forestry residues as well as livestock manure. It possesses superior characteristics such as a large specific surface area, adjustable pore architecture, abundant surface functional groups, and favorable electrical conductivity. With the increasingly severe global energy shortage and environmental pollution problems in recent years, biochar has emerged as a green, low-cost functional material with distinct application superiority in multiple key research directions, including energy storage and conversion, chemical catalysis, environmental restoration, and signal sensing and detection. This study comprehensively summarizes the latest research advances of biochar in the aforementioned application fields, focusing on innovative achievements in photocatalytic and electrocatalytic hydrogen generation, supercapacitors and electrochemical energy storage systems, persulfate activation technology, carbon dioxide capture, remediation of heavy metal and organic contaminants, volatile organic compound (VOC) adsorption, as well as electrochemical sensing devices. Existing research results demonstrate that modification strategies including metal and non-metal doping, surface oxidation treatment, and compounding with semiconductors or metal oxide materials can effectively improve the catalytic activity and functional performance of biochar. Furthermore, this paper prospects the future interdisciplinary development trends of biochar, analyzes the existing research gaps in mechanism exploration, structural optimization design, and industrial large-scale preparation, and provides theoretical and practical references for the further popularization and application of biochar in sustainable energy development and environmental governance fields.

## 1. Introduction

As energy consumption and environmental pollution become increasingly severe, the world has entered a critical window for energy transition and carbon neutrality. Despite rapid growth in clean energy investment and deployment in recent years [1,2,3,4,5,6,7,8,9,10,11,12,13,14,15,16,17], the global industry’s high dependence on traditional energy sources has kept carbon dioxide emissions at historically high levels with fluctuating upward trends, leading to coexisting climate risks and energy security concerns [18,19,20,21,22,23,24,25,26,27,28,29,30,31,32,33,34]. How to convert low-cost, renewable resources into high-value-added materials has become a key focus in sustainable development research. Against this backdrop, biochar as a carbon-rich porous material made from agricultural and forestry wastes exhibits unique versatility [35,36,37,38,39,40,41,42,43,44,45,46,47,48,49]. On the one hand, its high specific surface area, controllable pore structure, and abundant surface functional groups facilitate adsorption, anchoring of catalytic sites, and interfacial electron/proton transfer [50,51,52,53,54,55,56,57]. On the other hand, by stabilizing carbon in biomass and sequestering it in soil or material systems, biochar holds potential for long-term carbon sequestration [58,59,60,61,62,63].

In recent years, biochar has evolved from a traditional adsorbent material to a functional electrocatalytic/photocatalytic support and energy conversion enabler [64,65,66,67]. For example, biochar-assisted water electrolysis significantly reduces electrolysis energy consumption and improves process economics [68]. At the same time, when combined with semiconductors, biochar can enhance the separation of photogenerated charges and modify surface adsorption and reaction sites through interface engineering, thereby demonstrating excellent performance in processes such as photocatalytic hydrogen production, photogenerated H_2_O_2_ synthesis, and organic pollutant degradation [69,70,71,72,73,74,75,76].

Despite breakthroughs in these areas, existing literature is scattered across disciplinary subfields such as environmental remediation, energy materials, water treatment, and sensors [63,77,78,79,80,81]. There remains a lack of a cross-disciplinary review that integrates biochar’s mechanisms, materials, and processes in energy conversion, chemical catalysis, environmental remediation, and detection-particularly in three aspects: quantitative correlations between biochar’s physicochemical properties and its performance in different applications; interfacial electron/proton transfer mechanisms of biochar in low-energy hydrogen production and electrochemical/photocatalytic processes; and the large-scale preparation and life cycle assessment.

Building on the background provided, this paper reviews the most recent progress, underlying mechanisms, and potential applications of biochar across four main areas: energy, chemical engineering, environmental science, and detection. It draws from pertinent studies and recent significant experimental findings to offer researchers and engineers a comprehensive understanding of the current state of research and practical development directions.

## 2. Preparation and Modification Strategies of Biochar

### 2.1. Biochar Preparation Methods

Biochar is primarily produced via thermochemical conversion processes, using renewable biomass feedstocks such as agricultural/forestry wastes, livestock manure, sludge, and algae [82,83,84,85,86,87,88,89,90,91,92,93,94,95]. Common preparation methods include pyrolysis, hydrothermal carbonization (HTC) [96], gasification, and combined carbonization processes [97,98,99,100].

Pyrolysis is the most widely used method, typically conducted at 300–700 °C under oxygen-deficient conditions. Biochar from low-temperature pyrolysis retains abundant surface functional groups, making it suitable for environmental remediation; in contrast, high-temperature pyrolysis increases carbon content and graphitization degree, rendering it more appropriate for energy and electrochemical applications [101].

HTC operates in a high-pressure aqueous environment at 180–250 °C, ideal for high-moisture feedstocks. The resulting hydrochar has richer functional groups but relatively lower thermal stability [102].

Gasification is performed at 800–1000 °C, producing not only solid char but also syngas-offering dual value for energy generation and material production [103].

### 2.2. Biochar Modification Strategies

While raw biochar possesses inherent porosity and surface chemical activity, it often fails to meet the requirements of specific applications, necessitating further modification [104]. Common modification strategies are outlined below and Table 1:

Physical activation: This method uses CO_2_ or steam to etch pores at high temperatures, thereby increasing the specific surface area and micropore volume. It is green and non-toxic, though its ability to regulate pore structure is limited [105,106,107].

Chemical activation: Activators such as KOH, ZnCl_2_, or H_3_PO_4_ are added before or during carbonization. This significantly enhances the specific surface area and pore volume (up to 1000–2000 m^2^/g). However, extensive water washing is required to remove residual salts, which poses a risk of secondary pollution [108,109,110].

Surface functionalization: Biochar is treated with oxidants to introduce functional groups such as hydroxyls and carboxyls, strengthening its adsorption capacity for polar pollutants and metal ions. This approach is simple and efficient, but excessive oxidation may damage the pore structure [111].

Doping: Elements like Fe, Mn, Ni, Cu, N, S, or P are doped to adjust electron density, enhancing electrical conductivity and electrocatalytic activity. Notably, nitrogen doping effectively promotes electrochemical reactions and energy storage performance [112,113,114,115,116,117,118,119].

Composite modification: Biochar is combined with materials such as g-C_3_N_4_, TiO_2_, or conductive polymers to form composites [120,121,122]. This improves photoelectric properties and charge separation efficiency, expanding biochar’s application scope in photocatalysis, energy storage, and sensing.

**Table 1:** Comparison of biochar preparation and modification methods.

Method Category	Typical Conditions	Advantages	Disadvantages	References
Pyrolysis	300–700 °C, oxygen-deficient environment	Mature process, high yield, tunable properties	Requires dry feedstock, large performance variations	[98]
Hydrothermal Carbonization	180–250 °C, high-pressure aqueous phase	Suitable for wet feedstock, rich functional groups	Low thermal stability, activation required	[123]
Gasification	800–1000 °C, partial oxidation	Concurrent syngas and biochar production, high specific surface area	Low solid yield, complex control	[108]
Physical Activation	High-temperature treatment with CO_2_ or steam	Environmentally friendly, enhanced porosity	Limited pore structure regulation	[124]
Chemical Activation	Treatment with KOH, ZnCl_2_, H_3_PO_4_, etc.	Significantly increased specific surface area and pore volume	Requires water washing, cost and pollution issues	[125]
Surface Oxidation/Functionalization	Treatment with oxidants (e.g., HNO_3_, H_2_O_2_)	Enhanced polar adsorption, improved hydrophilicity	Excessive oxidation damages structure	[126]
Doping	Doping with Fe, Mn, N, S, P, et al.	Improved conductivity and reaction activity	Difficulty in controlling doping uniformity	[127,128]
Composite Modification	Composite formation with semiconductors or polymers	Enhanced photoelectric properties, expanded applications	Complex interface engineering, insufficient stability	[129]
Atmospheric-pressure microwave plasma pyrolysis	N_2_ plasma, atmospheric pressure; rapid direct heating	Rapid volumetric heating; no chemical activator; tunable surface chemistry and adsorption performance	Electricity demand, gas-flow control, limited scale-up and reactor-comparison data	[130]

### 2.3. Green Activation and a Transferable Structure–Function Framework

The preparation route determines which biochar functions are accessible. Feedstocks rich in lignin generally favor carbon yield and aromatic frameworks, whereas mineral-rich residues may provide intrinsic catalytic centers but can also block pores, as shown in Table 2. Increasing pyrolysis temperature usually removes oxygenated groups and increases aromaticity and conductivity; this benefits electron-transfer reactions but can weaken ion complexation and hydrophilicity. Activation increases accessible micro/mesoporosity, while N, S, or P doping redistributes charge density and metal/semiconductor hybridization introduces redox or photoactive sites. Consequently, no single biochar is universally optimal: high graphitization and connected mesopores are favored for high-rate electrodes; ultramicropores and low surface polarity favor CO_2_ capture; oxygenated or mineral sites favor metal-ion adsorption; and defect-rich sp^2^ domains coupled to redox centers favor persulfate activation and sensing.

Greener alternatives to conventional KOH, ZnCl_2_, or strong-acid activation include steam or CO_2_ activation, hydrothermal pretreatment of wet biomass, self-activation by naturally occurring alkali/alkaline-earth minerals, reusable salt templates, and activation with biomass-derived wood vinegar or pyrolysis liquids. These routes reduce corrosive reagent use and saline wash-water generation, but their pore-size control, yield, energy demand, and regeneration efficiency must be reported on a common basis. Atmospheric-pressure microwave plasma is an emerging rapid route: rice-straw and golden-shower-seed wastes were converted under N_2_ in 5 min, producing adsorption-active biochars without added chemical activator. The principal scale-up uncertainties are electricity intensity, plasma-gas demand, temperature uniformity, electrode/reactor lifetime, and continuous solids handling [130].

## 3. Application in the Field of Energy Materials

Electrochemical energy storage devices with high power and energy density are essential for lessening fossil fuel reliance and are crucial for the alternating storage of renewable energy [131,132,133,134,135,136,137,138,139,140,141,142]. Among diverse energy storage technologies, carbon stands out as a high-quality electrode material due to its abundant reserves, excellent conductivity, and precisely tunable intrinsic properties [143,144,145,146,147,148,149,150,151]. Biochar produced through pyrolysis and activation develops a multi-level pore structure, providing excellent pathways for the quick movement and storage of electrolyte ions in energy storage systems. The main energy benefit of biochar comes from its surface chemistry: a high concentration of oxygen-containing functional groups increases the adsorption of polar molecules and serves as active sites for electrochemical reactions. Additionally, high-temperature pyrolysis promotes graphitization, which enhances conductivity and supports efficient charge transfer.

Moreover, high-temperature pyrolysis promotes graphitization, thereby improving electronic conductivity and enabling efficient charge transport when used as an electrode material. When combined with metal oxides, carbon nitrides, or other semiconductors, biochar can form highly conductive networks that accelerate interfacial reaction kinetics, offering both structural and chemical support for energy conversion and storage [152,153]. At the mechanistic level, biochar can function as a catalyst support or co-catalyst, effectively facilitating electron and proton transfer during energy conversion processes [154].

### 3.1. Supercapacitors and Battery Devices

Within supercapacitor and battery systems, biochar serves dual critical functions: it not only enables physical energy storage but also imparts pseudo-capacitance via its surface functional groups or dopant elements [155]. Furthermore, it exerts a synergistic effect with metal oxides or sulfides, thereby boosting the specific capacitance and cycling stability of the entire energy storage system [156,157,158,159,160,161,162,163,164,165].

**Figure 1:**
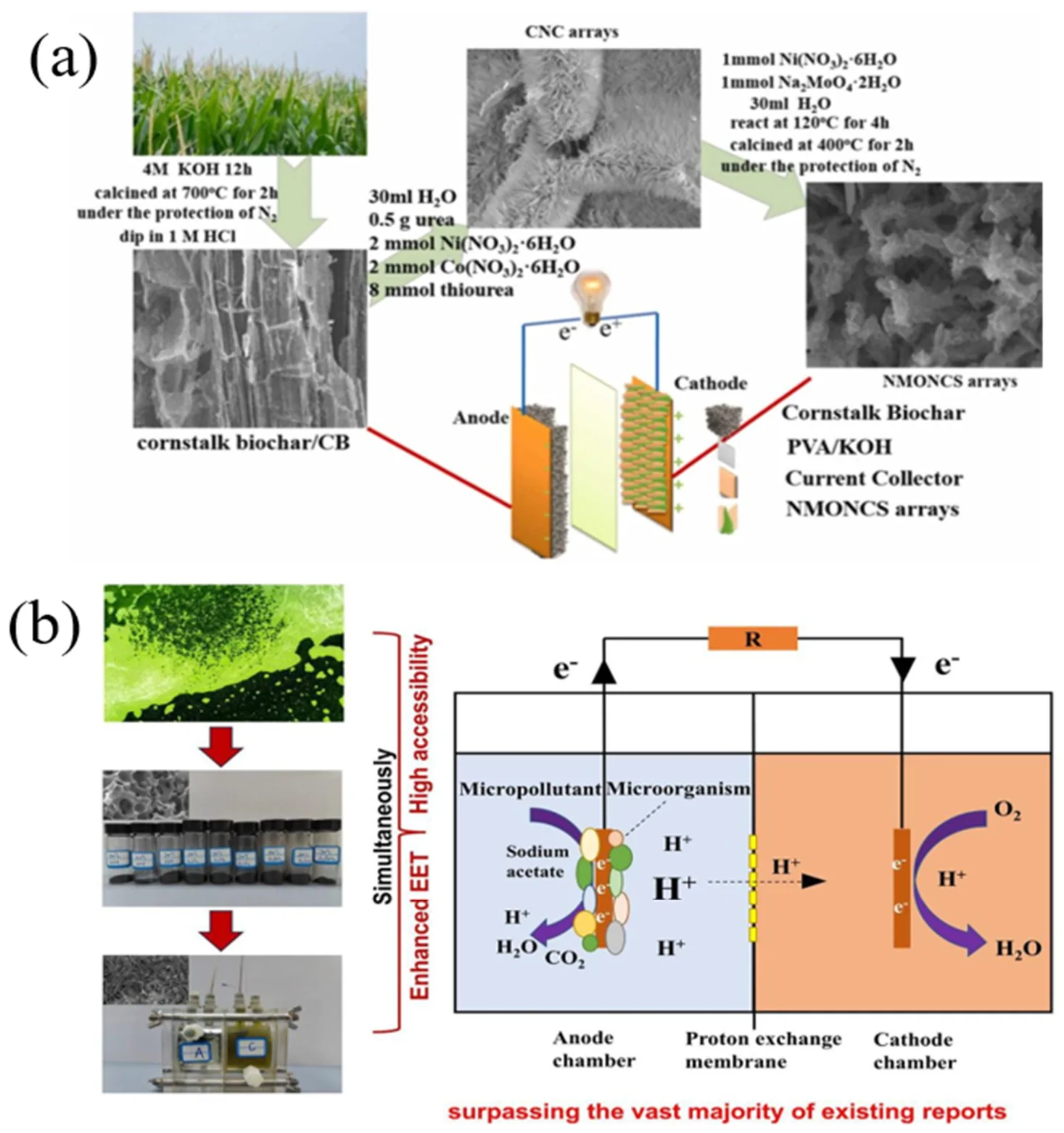
Biochar in electrochemical energy systems: (**a**) Hierarchically structured core-shell NiCo_2_S_4_@NiMoO_4_ nanoarrays anchored on cornstalk biochar serve as battery-type electrode materials for hybrid supercapacitor systems [166]; (**b**) Cyanobacteria-based biochar electrodes are utilized in microbial fuel cells to realize power generation and simultaneous pollutant removal [167].

Ren et al. proposed a sustainable strategy tailored for high-performance energy storage applications, as shown in Figure 1a [166]. Cornstalk-derived biochar (CB) was employed as a conductive scaffold to support NiCo_2_S_4_ nanoneedle arrays, which were further coated with NiMoO_4_ nanosheets-ultimately forming a hierarchical core-shell heterostructure. The as-fabricated NMONCS@CB electrodes exhibited remarkable electrochemical performance: an ultrahigh capacitance of 1447 F·g^−1^ at a current density of 5 mA·cm^−2^, paired with excellent long-term cycling stability (retaining 88% of the initial capacitance after 10,000 charge-discharge cycles). Moreover, the assembled asymmetric supercapacitor based on this electrode attained an energy density of 78.23 Wh·kg^−1^ at a power density of 184.49 W·kg^−1^, a performance that outperforms numerous previously reported NiCo_2_S_4_/NiMoO_4_-based systems. Notably, this device was capable of powering a red light-emitting diode (LED) continuously for 30 min. The superior performance of this system originates from its synergistic structural design: CB provides a highly porous architecture and excellent electrical conductivity, while the NiCo_2_S_4_@NiMoO_4_ heterostructure offers abundant redox-active sites and facilitates efficient charge transfer. Compared with conventional nickel foam substrates, CB not only reduces production costs and mitigates environmental impacts but also enhances the electrochemical utilization efficiency of the active materials.

Wang and the coauthors investigated the effect of cotton stalk particle size on the structural and electrochemical properties of derived biochar for lithium-ion battery anodes [168]. Biochar produced from larger particle sizes exhibited optimal tubular morphology, high interlayer spacing, and maximal pore volume, facilitating enhanced Li^+^ diffusion and specific capacity (271.7 mAh g^−1^ after 100 cycles at 0.1 A g^−1^). A critical particle size threshold of 200 μm was identified, below which structural properties deteriorated significantly. The study shows that managing the size of precursor particles is an energy-efficient and eco-friendly method to adjust the structure of biochar, allowing the production of high-performance anodes from agricultural waste without the need for chemical activation or doping.

### 3.2. Fuel Cells

Fuel cells are regarded as one of the most promising clean energy conversion technologies because they can directly convert chemical energy into electricity with high efficiency, low noise, and minimal pollutant emissions. Among various fuel cell systems, microbial fuel cells, hydrogen fuel cells, and direct alcohol fuel cells have attracted considerable attention for wastewater treatment, portable power supply, and distributed energy applications [169]. However, their practical development is still limited by sluggish electrode reaction kinetics, high catalyst cost, insufficient stability, and poor interfacial electron transfer. Biomass-derived carbon materials, especially biochar, offer a sustainable and low-cost alternative as electrode materials, catalyst supports, or conductive frameworks due to their porous structure, tunable surface chemistry, and good biocompatibility. Recent work has highlighted biochar as a promising electrode material in microbial fuel cells (MFCs), owing to its intrinsic porosity, conductivity, and surface chemistry [170,171,172,173].

In MFCs, biochar electrodes can facilitate extracellular electron transfer and reduce the ohmic resistance of the system, thereby enhancing power density. Zhang et al. demonstrate a K_2_CO_3_-modified cyanobacteria-derived biochar anode for microbial fuel cells, enabling synergistic bioelectricity generation and pollutant degradation as shown in Figure 1b [167]. The electrode achieves a 261.6-fold larger electrochemical active area than conventional carbon felt, reducing start-up time to 37.4 h with 693.04 mV voltage output, 1.076 W/m^2^ power density, 91.57% COD removal, and 47.41% Coulombic efficiency. It exhibits broad-spectrum degradation, including 95.12% tetracycline removal via pseudo-first-order kinetics involving hydroxylation, demethylation, deamination, and ring cleavage. Geobacter dominates the anode (52.48% abundance), with Comamonas as a key degrader (11.95%). At 0.3% the cost of Pt-C catalysts, this approach provides an economical and sustainable strategy for energy recovery and pollution control.

### 3.3. Photocatalytic and Electrochemical Conversion

Photocatalysis and electrocatalysis play pivotal roles in the development of clean energy technologies because they provide feasible pathways for converting abundant natural resources [174,175,176], such as solar energy, water, oxygen, nitrogen-containing pollutants, and carbon dioxide, into high-value fuels and chemicals under relatively mild conditions [177,178,179,180,181,182,183,184,185,186,187,188,189]. Biochar materials have attracted increasing attention in broader energy conversion processes, particularly in photocatalytic and electrochemical systems [190,191,192,193]. Their hierarchical porosity and tunable surface chemistry facilitate light absorption, interfacial charge separation, and electron/proton transfer [194,195,196,197,198,199]. Furthermore, strategies such as heteroatom doping and compositing with semiconductors or metal oxides significantly extend their applicability to key reactions, including water splitting, CO_2_ reduction, and organic pollutant degradation [200,201,202,203]. Understanding the mechanistic contributions of biochar and developing performance-optimization strategies are therefore crucial for advancing sustainable energy technologies.

**Figure 2:**
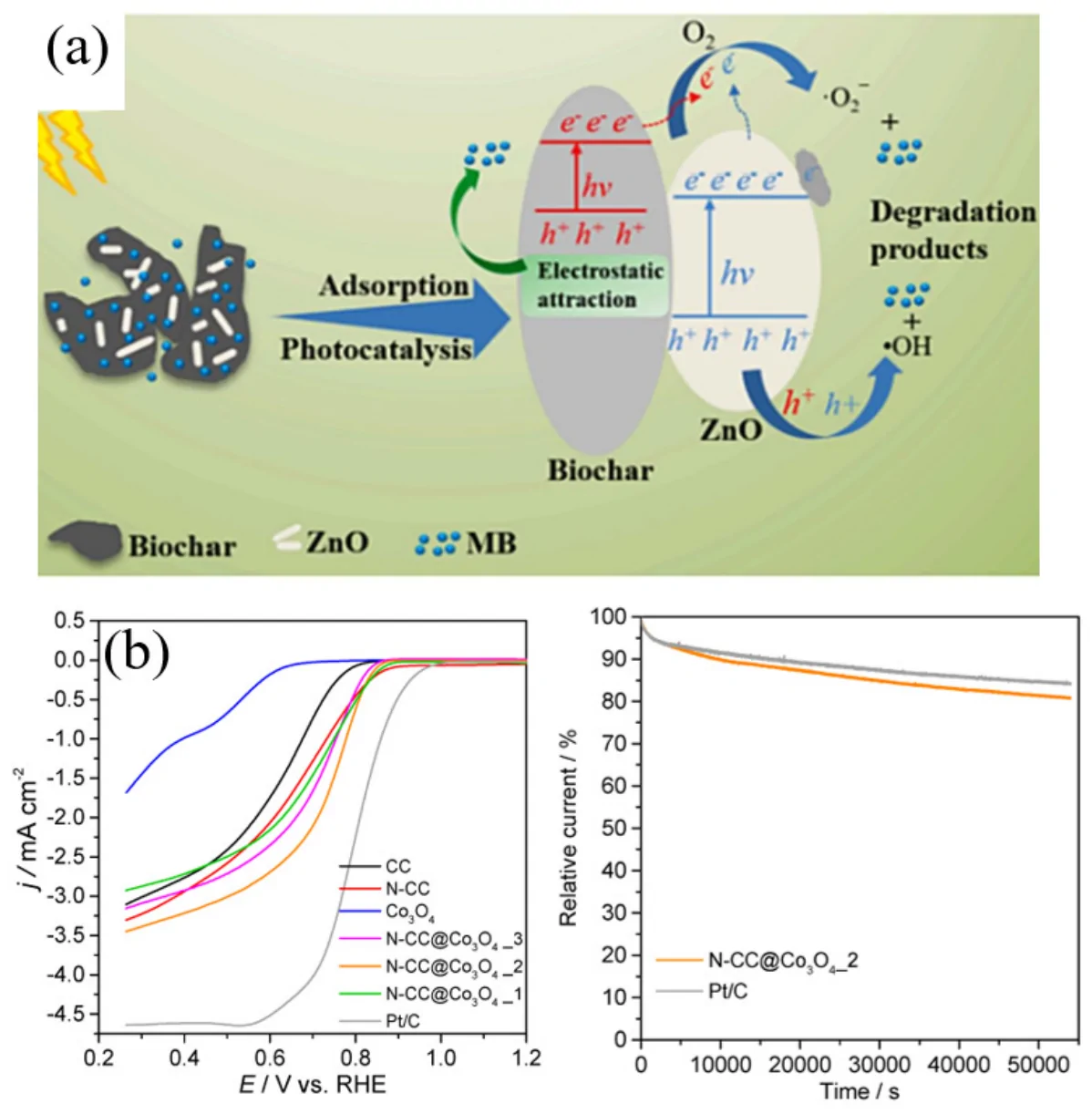
Biochar in catalytic energy conversion: (**a**) Biochar-Tailored g-C_3_N_4_ for piezo-photocatalytic H_2_O_2_ production [215]; (**b**) The left graph presents oxygen reduction reaction (ORR) polarization curves of the as-prepared electrocatalysts, while the right graph displays their electrochemical stability testing outcomes [214].

A major bottleneck in water electrolysis or fuel cells is the sluggish kinetics of the oxygen evolution reaction or oxygen reduction reaction [204,205,206,207,208,209,210,211,212,213]. This limitation can be alleviated by incorporating carbon-based materials derived from agricultural waste as low-cost reducing agents. Upon oxidative modification or metal/non-metal doping, biochar demonstrates enhanced surface reactivity and broader adaptability to electrochemical conditions. For instance, Matos and the coauthors present a sustainable ORR electrocatalyst comprising Co_3_O_4_ nanoparticles grown on nitrogen-doped shrimp shell biochar (N-CC) as shown in Figure 2b [214]. By optimizing the cobalt precursor loading, the N-CC@Co_3_O_4_ composite demonstrated superior performance, achieving an onset potential of 0.84 V, a limiting current density of −3.45 mA cm^−2^, and high selectivity for the 4-electron pathway. These results are attributed to the synergistic interaction between the optimized Co_3_O_4_ loading and the specific nitrogen configurations (pyridinic and graphitic N) within the porous support.

Parallel to electrochemical applications, biochar-supported photocatalysts (BSPs) have also gained momentum due to their superior performance in water splitting, hydrogen evolution [216]. For example, Chen and co-workers developed a biochar-modified carbon nitride (PCCN-10) containing nitrogen defects and carbon bridges, which exhibited outstanding piezo-photocatalytic activity for H_2_O_2_ production as shown in Figure 2a [215]. The modified material exhibits a significantly enhanced H_2_O_2_ generation rate of 4.61 mmol g^−1^ h^−1^ under combined light and ultrasonic irradiation without cocatalysts, outperforming most reported catalysts. Mechanistic studies revealed that nitrogen defects induced an asymmetric configuration that generated a strong dipole field, while carbon bridges enhanced π-π delocalization, together promoting spontaneous polarization and charge separation. The optimized photocatalyst also displayed excellent stability and was effective even in pure water, achieving a rate of 2.19 mmol g^−1^ h^−1^. This work highlights defect engineering and dipole field modulation as promising strategies for designing multifunctional, biochar-based photocatalysts for sustainable energy conversion. Mechanistic studies revealed that nitrogen defects induced an asymmetric configuration that generated a strong dipole field, while carbon bridges enhanced π-π delocalization, together promoting spontaneous polarization and charge separation. The optimized photocatalyst also displayed excellent stability and was effective even in pure water, achieving a rate of 2.19 mmol g^−1^ h^−1^. This work highlights defect engineering and dipole field modulation as promising strategies for designing multifunctional, biochar-based photocatalysts for sustainable energy conversion.

### 3.4. Cross-Study Quantitative Synthesis and Remaining Energy Limitations

Across the energy studies summarized here, performance improves when preparation controls both transport and active-site utilization rather than maximizing surface area alone. The cornstalk scaffold/heterostructure delivered 1447 F g^−1^ and 88% retention after 10,000 cycles, whereas particle-size-controlled cotton-stalk biochar reached 271.7 mAh g^−1^ after 100 cycles. In microbial fuel cells, K_2_CO_3_ modification enlarged the electrochemically active area by 261.6-fold and produced 1.076 W m^−2^. These values are not pooled into a single effect size because device configuration, mass loading, electrolyte, potential window, and reporting basis differ; instead, Table 3 provides a structured evidence matrix. Future studies should report raw-biochar and commercial-carbon controls, yield, ash content, BET/micropore area, conductivity, mass loading, and full-cell metrics to enable a defensible meta-analysis.

## 4. Application in the Environmental Field

In the field of environmental remediation, biochar exhibits significant advantages due to its unique physicochemical properties [217,218,219,220,221,222,223,224,225,226]. Its high specific surface area and well-developed porous structure provide abundant active sites for the physical adsorption of pollutants, enabling effective capture of heavy metal ions, persistent organic pollutants, and dyes in water and soil [227,228,229,230,231]. Additionally, the oxygen-containing functional groups and mineral components on the surface of biochar further enhance its capacity for ion complexation and electrostatic adsorption, allowing it to maintain high purification efficiency even in diverse and complex environmental media.

### 4.1. Persulfate Activation

Advanced oxidation processes (AOPs) are considered highly effective for the degradation of persistent organic pollutants, but their efficiency largely depends on the rapid activation of oxidants [232,233]. Biochar, when modified or combined with transition metals, can serve as an efficient activator by promoting electron transfer and generating reactive species. The biochar-based composites provide a low-cost and sustainable pathway for enhancing persulfate activation and pollutant degradation [234,235,236,237].

**Figure 3:**
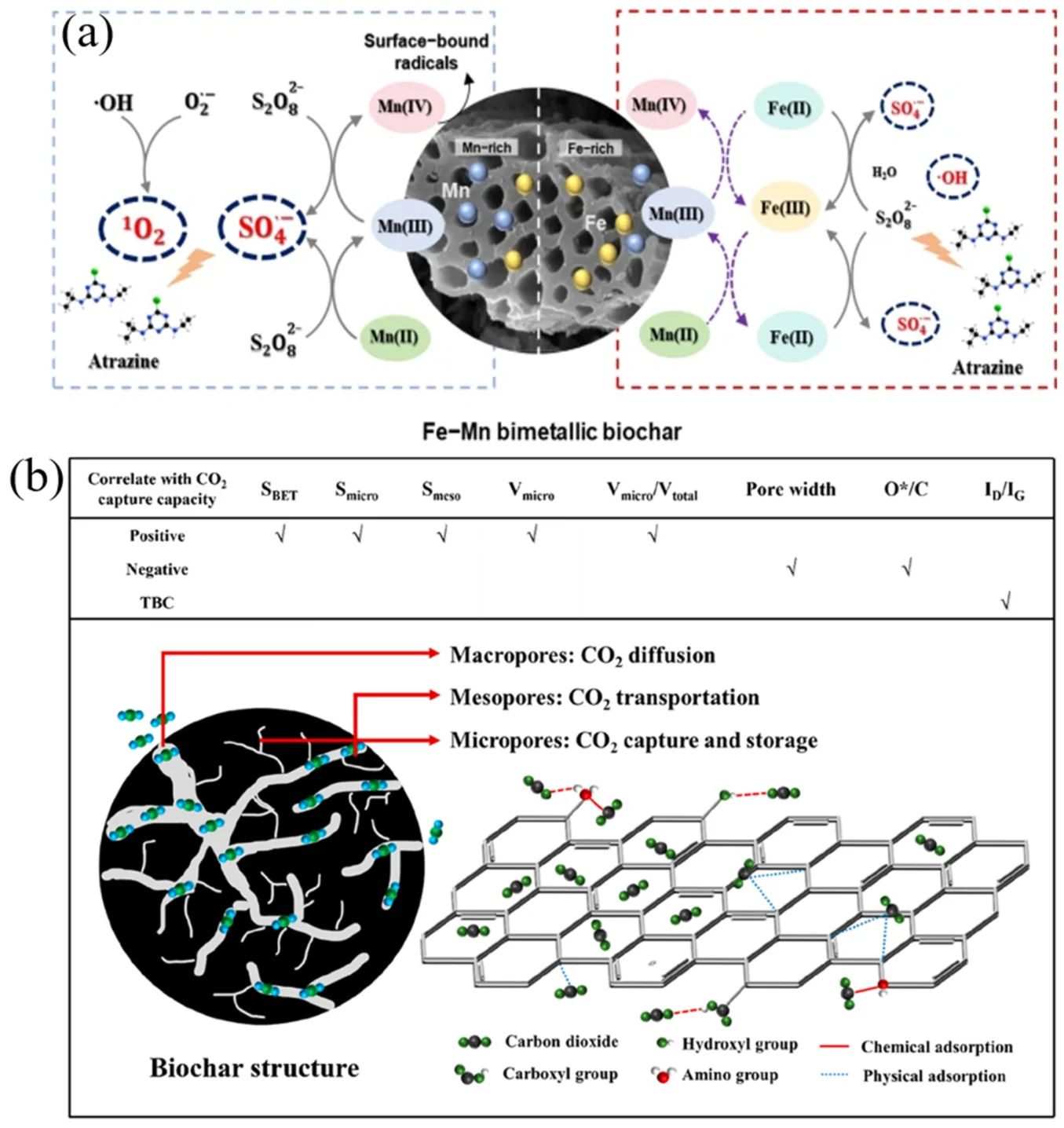
Environmental mechanisms: (**a**) Bimetallic biochar composites incorporating iron and manganese elements are adopted to efficiently trigger persulfate activation [238]; (**b**) The potential mechanism underlying physical adsorption of CO_2_ on porous biochar materials is investigated [108].

In Liang’s research, iron-manganese bimetallic biochar composites were used to significantly enhance persulfate activation for efficient atrazine degradation, with Fe-rich variants outperforming Mn-rich and monometallic counterparts, as shown in Figure 3a [238]. The Fe-biochar/PS system achieved exceptional atrazine removal rates of 96.7% primarily through free radical pathways (SO_4_•^−^ and •OH), while Mn-rich systems involved both radical and non-radical mechanisms. The superior performance of Fe-rich biochar is attributed to its enhanced electron transfer capacity, higher specific surface area, and synergistic valence exchange between Fe^2+^/Fe^3+^ and Mn^3+^/Mn^4+^ that facilitates continuous persulfate activation. Degradation pathways involved alkyl hydroxylation/oxidation, dealkylation, and dechlorohydroxylation, ultimately yielding less toxic cyanuric acid. These findings provide critical insights for designing efficient carbon-metal composites for advanced oxidation processes in environmental remediation.

### 4.2. CO_2_ Capture

The rising atmospheric CO_2_ concentration poses significant challenges to climate change mitigation, highlighting the urgent need for efficient and sustainable carbon capture technologies [239,240,241,242,243,244,245,246,247,248,249,250,251,252]. Owing to its large surface area, tunable porosity, and adjustable surface polarity, biochar is increasingly recognized as a promising CO_2_ adsorbent [253,254,255,256]. Modification strategies such as physical/chemical activation and heteroatom doping further enhance its adsorption capacity, making biochar a viable candidate for post-combustion carbon capture [257].

Zhang and the coauthors systematically evaluated three lignocellulosic biochars activated via physical and chemical methods for post-combustion CO_2_ capture as shown in Figure 3b [108]. KOH-activated biochar achieved the highest capacity exceeding 2.35 mmol/g at 1 bar and 298 K, attributed to its significantly enhanced surface area and microporous volume. Multi-method characterization revealed that CO_2_ adsorption capacity is positively correlated with specific surface area and micropore volume but negatively influenced by pore width and surface oxygen content (O*/C ratio). The research demonstrates that while chemical activation generally outperforms physical methods, the choice of activator is critical, as some agents like H_3_PO_4_ can reduce adsorption performance. These findings provide essential design principles for developing highly efficient, biochar-based adsorbents tailored for carbon capture applications, emphasizing the synergy between high surface area, optimized micro-porosity, and low surface polarity.

### 4.3. Heavy Metal Pollution Remediation

Heavy metal contamination in water and soil remains a pressing environmental issue due to the persistence, bioaccumulation, and toxicity of metal ions, such as Pb^2+^, Cd^2+^, and As^5+^ [258,259,260,261,262,263,264,265,266,267,268,269,270,271]. The surface functional groups and mineral components in biochar enable complexation, ion exchange, and precipitation mechanisms, which contribute to efficient heavy metal immobilization. Moreover, hybrid biochar composites integrate adsorption and catalytic functions, offering multifunctional solutions for heavy metal remediation [272,273].

**Figure 4:**
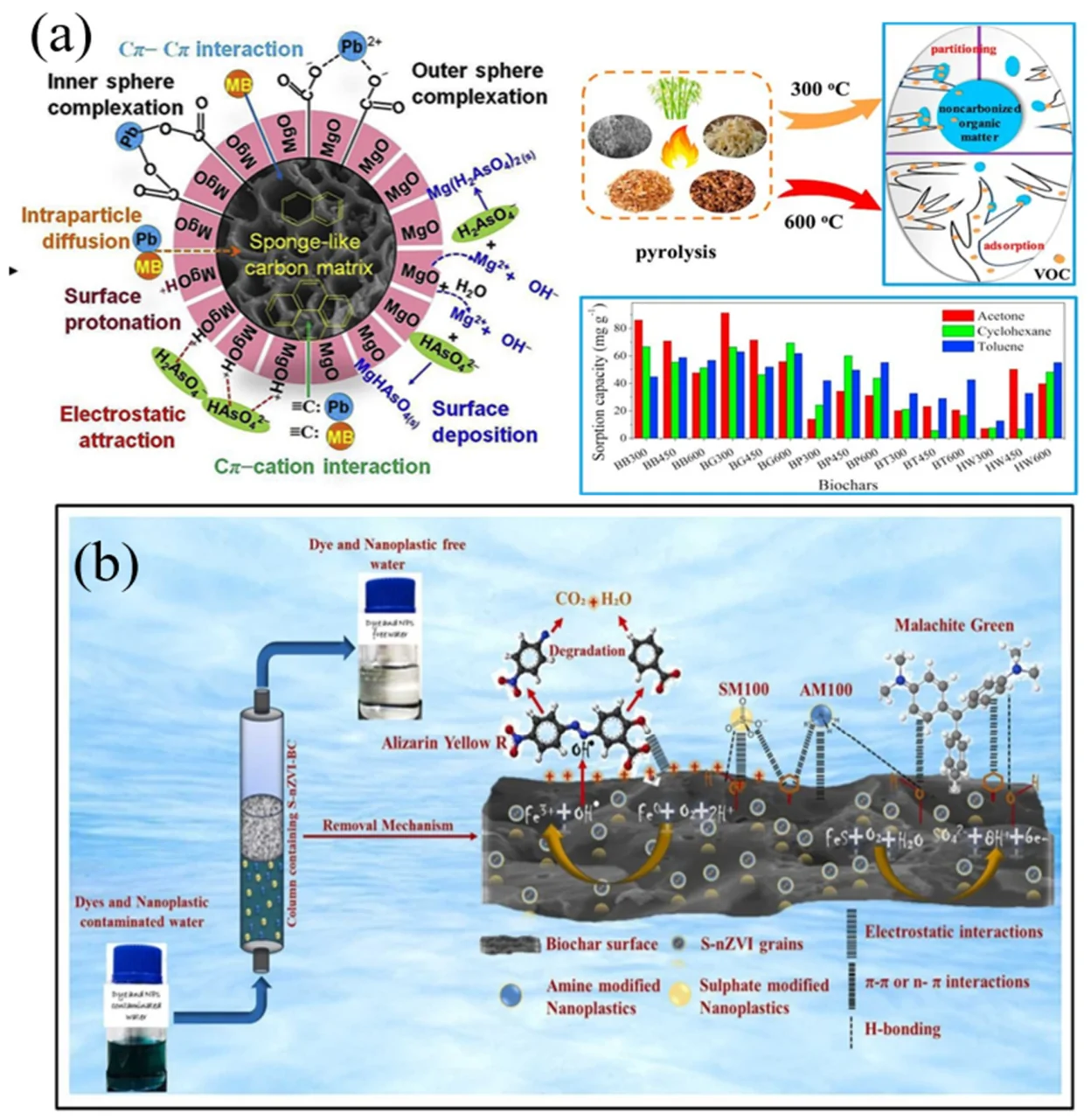
Pollutant removal by modified biochar: (**a**) MgO-modified sponge-like carbon composites can effectively capture arsenic (V), lead (II), and methylene blue contaminants from water solutions [275]; (**b**) Biochar materials are applied for the elimination of volatile organic compounds (VOCs) [276].

Heavy metal pollution from industrial activities poses severe threats to ecosystems and human health due to the persistence, toxicity, and bioaccumulation of contaminants like arsenic and lead in water and soil [274]. To address this, Li et al. developed a novel MgO hybrid sponge-like carbonaceous composite (HSC) derived from sugarcane leafy trash via an integrated adsorption-pyrolysis method as shown in Figure 4a [275]. This composite exhibited a unique structure combining nano-sized MgO flakes with a nanotube-like carbon sponge, significantly enhancing its adsorption capabilities. The HSC demonstrated exceptional maximum adsorption capacities of 157 mg/g for As^5+^, 103 mg/g for Pb^2+^, and 297 mg/g for methylene blue (MB), outperforming many conventional adsorbents. Mechanistic studies revealed that As^5+^ removal was primarily governed by surface deposition forming MgHAsO_4_ and Mg(H_2_AsO_4_)_2_ crystals, while Pb^2+^ and MB adsorption involved π-π interactions and complexation with functional groups on the carbon matrix. The hybridization process not only improved structural properties but also offered a cost-effective production method, estimated at $0.54/kg, making it a promising, sustainable alternative to commercial activated carbon for efficient multi-pollutant wastewater remediation.

### 4.4. Control of Volatile Organic Compounds

Volatile organic compounds (VOCs) are a major class of atmospheric pollutants, contributing to photochemical smog formation and posing health risks [277,278,279,280,281,282,283,284,285,286,287,288,289,290,291,292,293]. Traditional adsorbents such as activated carbon are effective but often costly and less sustainable [294]. Biochar, derived from diverse feedstocks under controlled pyrolysis conditions, provides a low-cost, renewable alternative with strong VOC sorption capacity. Its adsorption performance is closely linked to feedstock properties, pyrolysis temperature, and the balance between surface area and organic matter content.

In Zhang’s study, the sorption performance and mechanisms of biochar for removing VOCs from the gas phase were investigated, utilizing fifteen biochars produced from five distinct feedstocks at three pyrolysis temperatures as shown in Figure 4b [276]. The results demonstrate that all biochars exhibit significant VOC sorption capacities, ranging from 5.58 to 91.16 mg g^−1^ for acetone, cyclohexane, and toluene. The sorption performance is governed by a dual mechanism: physical adsorption, which dominates in high-temperature biochars with larger surface areas, and partition into noncarbonized organic matter (NOM), which prevails in low-temperature biochars with higher NOM content. The process is exothermic, with sorption capacities decreasing as system temperature increases. Notably, biochars maintain excellent reusability, showing only a 6.9–14.7% reduction in capacity after five consecutive sorption-desorption cycles. This work establishes biochar as a highly promising, cost-effective alternative to conventional sorbents like activated carbon for efficient gaseous VOC removal, with performance intricately linked to both feedstock selection and pyrolysis conditions.

### 4.5. Microplastic and Nanoplastic Remediation

Microplastics and nanoplastics have emerged as persistent environmental contaminants due to their widespread distribution, small particle size, high chemical stability, and potential ecological toxicity [295,296,297]. They can accumulate in aquatic and terrestrial environments, adsorb coexisting pollutants such as heavy metals and antibiotics, and enter food chains, posing risks to ecosystems and human health. Therefore, developing sustainable and efficient remediation strategies is urgently needed. Biomass-derived carbon materials, especially biochar, have attracted increasing attention for microplastic and nanoplastic removal because of their porous structure, large surface area, tunable surface functional groups, and low-cost renewable origin. Through hydrophobic interaction, electrostatic attraction, pore interception, π-π interaction, and surface complexation, biochar can effectively capture plastic particles and associated pollutants. Moreover, modified biochar can be integrated with photocatalytic, electrocatalytic, or advanced oxidation systems to promote plastic surface oxidation and partial degradation, providing a promising route for microplastic remediation.

Ganie and the coauthors presented the development of a sustainable composite adsorbent, Sulfidised nano-Zerovalent Iron embedded in Biochar (S-nZVI-BC), synthesized from agro-waste sugarcane bagasse [298]. The material was engineered for the simultaneous removal of organic dyes and microplastics from wastewater. The research optimized the Sulfur/Iron molar ratio to 0.3, achieving a material with high surface area and reactivity. The composite demonstrated exceptional performance in both batch sorption and continuous column filtration modes, removing over 90% of contaminants in less than 15 min. The primary removal mechanisms were identified as degradation, electrostatic attraction, complexation, and π−π interactions.

### 4.6. Biochar-Based Solar Interfacial Evaporation for Seawater Desalination

**Figure 5:**
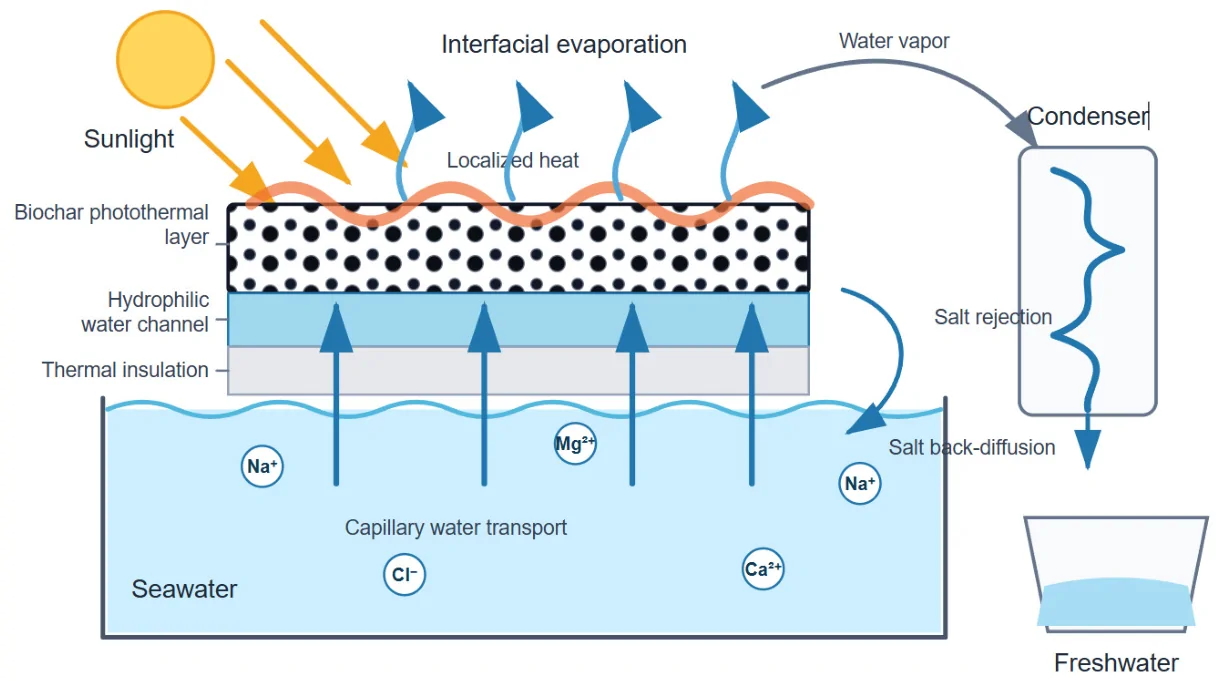
Schematic diagram Biochar-Based Solar Interfacial Evaporation for Seawater Desalination.

Freshwater scarcity has stimulated the development of low-energy desalination technologies. Solar-driven interfacial evaporation localizes solar heat at the air–water interface rather than heating the entire bulk solution, thereby reducing conductive heat loss and improving solar-energy utilization [299,300,301,302]. Biochar is a promising photothermal material for this application because its conjugated carbon domains provide broadband solar absorption and efficient light-to-heat conversion, while its hierarchical pore structure, hydrophilic functional groups, low density, and renewable origin facilitate water transport, vapor escape, and floating evaporator construction, as shown in Figure 5. Compared with noble-metal, semiconductor, and synthetic carbon absorbers, biochar-based evaporators can also reduce material cost and valorize agricultural, forestry, algal, and industrial biomass residues.

The performance of a biochar solar evaporator is governed by the coordinated regulation of light absorption, water supply, thermal localization, and vapor transport. Aromatic and graphitized carbon domains absorb ultraviolet, visible, and near-infrared radiation and dissipate the absorbed energy as heat. Meanwhile, oxygen- and nitrogen-containing functional groups improve wettability and continuously transport water to the heated interface through capillary action. Interconnected macropores provide water-supply channels, whereas mesopores and micropores increase the evaporation area and facilitate vapor diffusion. Thermal-insulating supports, including cellulose sponges, polymer foams, and aerogels, suppress heat conduction from the evaporation layer to bulk seawater. However, excessive hydrophilicity may increase the amount of water that must be heated, while an overly thick biochar layer may restrict water and vapor transport. Therefore, high solar absorption alone is insufficient; the absorber thickness, wettability, pore connectivity, thermal conductivity, and water-supply rate must be optimized simultaneously.

Representative studies demonstrate the feasibility of converting different biomass wastes into solar evaporators. A hydrophilic porous carbon membrane prepared from willow catkins achieved approximately 96% solar absorption, an evaporation rate of 1.65 kg m^−2^ h^−1^, and a solar-to-vapor conversion efficiency of approximately 90.5% under one-sun irradiation [303]. It also removed 99.9% of sodium ions from actual seawater, demonstrating the combined effects of hierarchical porosity, capillary water transport, and interfacial heat localization. A microalgae-residue biochar evaporator exhibited a light-absorption efficiency of 94.1% and an evaporation rate of 1.165 kg m^−2^ h^−1^, illustrating the possibility of integrating biodiesel-residue utilization with freshwater production. Carbonized sugarcane produced an evaporation rate of 1.69 kg m^−2^ h^−1^, and an efficiency of 85% under one sun, together with an average ion rejection of 99.32%. More recently, CaCO_3_-activated porous carbon derived from lignosulfonate was incorporated into a poly(vinyl alcohol) matrix. The resulting PVA@PCLS evaporator exhibited more than 97% absorption over 250–2500 nm, an evaporation rate of 2.33 kg m^−2^ h^−1^, and a solar-to-vapor efficiency of 83.7% [304]. These results indicate that feedstock-derived pore architecture and evaporator-level thermal management are equally important for desalination performance.

Despite these advantages, salt accumulation remains a major obstacle to long-term seawater desalination. As water evaporates, salts can crystallize on the photothermal surface, block pores, reduce light absorption, and interrupt water transport. Salt resistance can be improved by constructing interconnected water channels that promote ion back-diffusion, introducing hydrophilic gradients, limiting the contact area between the evaporator and bulk water, or designing self-cleaning and periodically regenerable surfaces. Future studies should evaluate biochar evaporators using actual seawater or concentrated brine rather than only deionized water and should report dark evaporation, net evaporation rate, solar-to-vapor efficiency, salt rejection, condensate quality, outdoor water yield, cycling stability, biochar leaching, and resistance to biological fouling. Life-cycle and techno-economic analyses should also include feedstock collection, pyrolysis energy, chemical activation, washing, supporting substrates, condensation systems, evaporator lifetime, and end-of-life disposal. These considerations are essential for translating laboratory-scale evaporation rates into reliable freshwater production under practical outdoor conditions.

### 4.7. Mechanism Comparison and Cross-Study Quantitative Synthesis

Environmental performance reflects the competition among adsorption, radical oxidation, and surface-mediated non-radical electron transfer, as presented in Table 4. Adsorption dominates when pore size matches the target and surface groups provide electrostatic attraction, complexation, hydrogen bonding, or π-π interactions. Radical persulfate activation produces SO_4_•^−^ and •OH and is rapid but vulnerable to scavengers and matrix constituents. Non-radical pathways-including surface-bound radicals, singlet oxygen, and direct electron transfer-are often more selective and salt-resistant but require conductive defect domains and appropriately cycled metal centers. ROS assignment should therefore combine quenching, electron-paramagnetic-resonance evidence, electrochemical tests, and mass balance rather than rely on a single scavenger experiment. For CO_2_, ultramicropore volume and low surface polarity are generally more decisive than total BET area; for persulfate, electron-donating defects, graphitic conductivity, and reversible Fe/Mn redox couples are more important than surface area alone.

## 5. Application of Detection and Sensing

With the growing demand for rapid, sensitive, and cost-effective analytical technologies, detection and sensing systems have become increasingly important in environmental monitoring, food safety, biomedical diagnosis, and industrial process control, as presented in Table 5 [305,306,307,308,309,310,311,312,313,314,315,316,317,318,319,320,321,322,323,324]. Conventional sensing materials often rely on noble metals, complex nanostructures, or expensive synthetic carbon materials, which may limit their large-scale application [325,326,327,328,329,330,331,332,333,334,335,336,337,338,339,340,341,342,343,344,345,346,347]. In this context, biomass-derived carbon materials, especially biochar, provide a sustainable and versatile platform for sensor construction [325,348,349,350,351,352]. Their hierarchical porous structure can enrich target analytes, while tunable surface functional groups, heteroatom dopants, and defect sites can enhance electron transfer and interfacial recognition. Moreover, biochar can be easily combined with metal oxides, conductive polymers, enzymes, or two-dimensional materials to improve sensitivity, selectivity, and stability. Therefore, biochar-based sensing materials show great potential for detecting heavy metals, pesticides, organic pollutants, gases, and biological molecules in complex real samples. Conventional sensors often rely on costly noble metals or fragile biomolecules, which limit large-scale deployment [313,353,354,355,356]. Biochar, as a biomass-derived porous carbon material, offers a promising alternative. Its adjustable conductivity, diverse surface functionalities, and strong compatibility with nanomaterials allow the design of sensors with high sensitivity and selectivity, while its renewable origin and low production cost meet the requirements of scalable and eco-friendly applications.

**Figure 6:**
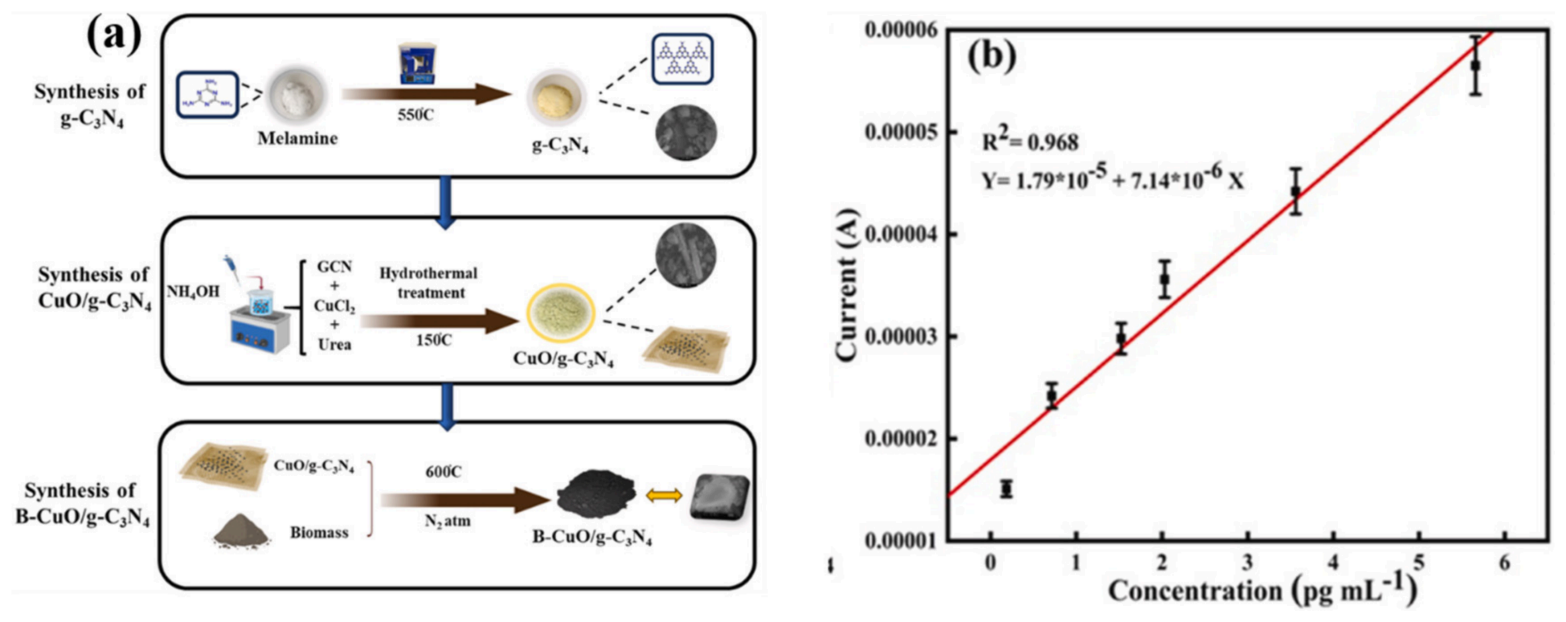
(**a**) Schematic illustration for the synthetic route of B-CuO/g-C_3_N_4_ composites, (**b**) Calibration curve established within the MALA concentration range of 0.184–5.660 pg mL^−1^ [357].

As shown in Figure 6a,b, Devi et al. prepared a novel non-enzymatic electrochemical sensor utilizing a ternary nanocomposite of biochar-supported copper oxide and graphitic carbon nitride (B-CuO/g-C_3_N_4_) for the ultrasensitive detection of the organophosphorus pesticide malathion (MALA) [357]. Synthesized via a hydrothermal method followed by calcination, the nanocomposite exhibited exceptional electrochemical properties, including a high electrochemically active surface area, low charge transfer resistance, and strong π-π interactions that enhance charge separation and MALA complexation. Employing square wave anodic stripping voltammetry (SWASV), the sensor demonstrated a remarkably low limit of detection (1.2 pg mL^−1^) and a wide linear range (0.184–5.660 pg mL^−1^). The sensing mechanism is attributed to the synergistic effect of physical adsorption on the porous biochar and the redox activity of CuO, facilitating MALA oxidation. The sensor showed excellent reproducibility, repeatability, and anti-interference capability, with successful application in detecting MALA in spiked apple and tomato samples, achieving recoveries of 87.64–120.59%. This work establishes B-CuO/g-C_3_N_4_ as a highly promising, cost-effective platform for non-enzymatic pesticide monitoring, offering significant advantages over traditional enzyme-based sensors in stability and practical deployment.

Yang and the coauthors achieved the synthesis of nitrogen-doped biochar (N-BC) derived from waste mushroom bran via a hydrothermal and activation-carbonation strategy for electrochemical detection of Pb^2+^ and Cd^2+^ [358]. The N-BC3.5 material exhibited optimal performance with a high specific surface area (154.56 m^2^/g), enriched surface functional groups, and improved electrical conductivity due to successful nitrogen doping. The constructed N-BC3.5/GCE sensor demonstrated an excellent electrochemical response under optimized conditions, achieving low detection limits of 0.0615 μM for Pb^2+^ and 0.118 μM for Cd^2+^ with high sensitivity and a wide linear range. The sensor facilitated both individual and simultaneous detection of these heavy metal ions, showcasing good stability and reusability over multiple cycles. This work highlights the potential of waste-derived, nitrogen-doped biochar as an efficient, eco-friendly sensing material for advanced electrochemical monitoring of toxic pollutants in environmental applications.

## 6. Scalability, Techno-Economics, Life-Cycle Considerations, and Durability

Scale-up must treat feedstock variability as a controlled input rather than an unavoidable nuisance. At minimum, studies should report moisture, ash, volatile matter, elemental composition, particle size, seasonal source, char yield, energy input, wash-water volume, and mass loss during activation, as presented in Table 6. Blending, pelletization, in-line moisture control, and acceptance windows for ash and H/C and O/C ratios can reduce batch variability. Continuous auger, rotary-kiln, and fluidized-bed reactors improve throughput, whereas microwave/plasma routes offer rapid heating but require transparent electricity and gas balances. Reproducibility should be demonstrated across at least three independently produced batches, not only replicate measurements from one batch.

The available economic evidence in the reviewed studies is promising but not yet directly comparable. The MgO-hybrid sponge-like carbon was estimated at US$0.54 kg^−1^, and the modified microbial-fuel-cell electrode was reported at 0.3% of the cost of Pt/C; these estimates use different boundaries and should not be interpreted as a universal cost advantage. Compared with nickel foam, biochar can replace a mined metal current collector with a renewable porous scaffold, but hybrid sulfide/oxide deposition, binder use, and durability may dominate impacts. Compared with commercial activated carbon, biochar may avoid fossil precursors and provide carbon-storage credit, yet high-temperature heating, KOH production, washing, wastewater neutralization, drying, and lower regeneration life can reverse that advantage. A defensible comparison, therefore, requires a functional unit (for example, 1 kWh delivered over device life or 1 kg pollutant removed), cradle-to-grave boundaries, allocation of biomass-residue burdens, regional electricity mix, transport, chemical recovery, regeneration, leaching, end-of-life, and uncertainty/sensitivity analysis.

Regeneration and long-term stability remain application-specific bottlenecks. Adsorbents should be evaluated through multiple adsorption–desorption cycles with capacity retention, desorbate management, and metal leaching. Catalysts require total organic carbon/mineralization, intermediate toxicity, and ion-leaching measurements. Electrodes require full-cell cycling at practical mass loading. Plasma and chemical activation should be compared using energy per kilogram of saleable char, char yield, water use, and hazardous-effluent intensity. These reporting requirements integrate environmental and economic performance into the thematic interpretation rather than treating sustainability as an untested intrinsic property.

## 7. Theoretical and Computational Studies

Computational methods can connect preparation, atomic structure, and macroscopic performance [359,360,361]. Density functional theory can compare adsorption energies, charge-density differences, and reaction barriers on graphitic edges, vacancies, heteroatom sites, and metal–biochar interfaces; molecular dynamics can evaluate ion diffusion and competitive adsorption in solvated pores; pore-network and continuum models can separate intrinsic kinetics from transport limitations; and machine-learning models can map feedstock composition and pyrolysis conditions to yield, H/C and O/C ratios, surface area, conductivity, or application metrics. Computation should be constrained by experimentally measured ash/mineral phases, pore-size distributions, XPS-derived site populations, and realistic aqueous speciation. Prospective validation—predicting an unseen feedstock or synthesis condition before experiment—is needed to prevent overfitting and to convert correlation into design rules.

## 8. Summary and Outlook

To summarize, as a porous carbonaceous material derived from renewable biomass, biochar features high specific surface area, tailorable porous architecture, abundant surface functional moieties, and strong environmental sustainability-underpinning its vast application potential in energy storage/conversion, chemical engineering, environmental remediation, and chemo/biosensing. In energy, it acts as a cost-effective electrode for devices like lithium-ion batteries and supercapacitors, while heteroatom doping or semiconductor compositing boosts its efficiency in photocatalytic/electrocatalytic HER In chemical engineering, it serves as a versatile support: dispersing metal nanoparticles, facilitating interfacial electron transfer, and delivering excellent performance in green synthesis and AOPs for organic pollutant mineralization. In environmental remediation, it sequesters heavy metals, persistent organic pollutants, and gaseous pollutants via physisorption, complexation, and free radical degradation, while aiding carbon sequestration for climate mitigation. In sensing, its tunable conductivity, diverse surface chemistry, and compatibility with sensitive elements make it a top candidate for green, low-cost, high-sensitivity chemo/biosensors.

However, biochar’s scalable application and industrialization face key challenges. First, its properties depend heavily on feedstock composition and pyrolysis/activation parameters, causing batch-to-batch variations-demanding standardized, controllable fabrication protocols. Second, chemical activation and doping enhance functionality but bring high energy use, hazardous reagents, and secondary pollution; future research should prioritize eco-friendly modification. Third, hybridization with materials like metal oxides or g-C_3_N_4_ is often needed for energy/catalysis, but balancing performance, structural integrity, and cyclic stability remains unresolved.

At the application level, the development of biochar should be consistent with goals of carbon neutrality and the circular economy. Utilizing large quantities of agricultural residues and municipal sludge to produce biochar enables both waste recovery and carbon emission reduction. Additionally, conducting comprehensive cradle-to-grave life cycle assessments is crucial to measure energy consumption, carbon footprint, and ecotoxicity, thereby ensuring genuine sustainability. Importantly, advanced characterization techniques and simulations can reveal the relationships between nanoscale structures and performance, helping to create models that link structure, function, and application, which guide precise material design. Combining artificial intelligence with high-throughput experiments will further speed up the screening of feedstocks and production methods, facilitating the transition of biochar from laboratory research to industrial-scale implementation.

In conclusion, biochar-versatile and eco-friendly-has great prospects in energy conversion, green catalysis, remediation, and sensing. Future research must break through in green scalable fabrication, precise performance control, and large-scale validation to turn it from a prospective material to a core functional material. As global energy transition advances, biochar will play an irreplaceable role in achieving dual carbon goals and building a low-carbon energy system.

## Figures and Tables

**Table 2:** Cross-application structure–function–performance framework.

Material Descriptor	Dominant Interfacial Role	Applications Favored	Trade-Off/ Limitation
Micropore volume (<2 nm)	Confinement and high adsorption potential	CO_2_ capture; small-molecule adsorption	Slow diffusion or pore blockage at high loading
Connected mesopores/macropores	Ion/mass-transport highways	High-rate energy storage; flow-through remediation	Lower gravimetric surface area if overdeveloped
O-containing groups	Complexation, H-bonding, wettability, pseudocapacitance	Metal adsorption; aqueous sensing; capacitors	Reduced conductivity and thermal stability
Graphitic sp^2^ domains/defects	Electron transfer and π interactions	Electrocatalysis; persulfate activation; sensing	High temperature may remove useful polar sites
N/S/P dopants	Charge redistribution and chemically selective binding	ORR/OER; batteries; electrochemical sensing	Dopant identity and uniformity are difficult to compare
Metal/oxide/semiconductor interface	Redox cycling, charge separation, catalytic sites	AOPs; photocatalysis; hybrid electrodes	Leaching, aggregation, and long-term stability

**Table 3:** Quantitative comparison of representative energy applications.

Feedstock/ System	Modification/ Design	Standardized Metric	Mechanistic Interpretation	Ref.
Cornstalk biochar	NiCo_2_S_4_@NiMoO_4_ core–shell arrays	1447 F g^−1^; 88% after 10,000 cycles; 78.23 Wh kg^−1^	Conductive porous scaffold plus abundant redox sites	[166]
Cotton stalk	Particle-size-controlled pyrolysis	271.7 mAh g^−1^ after 100 cycles at 0.1 A g^−1^	Tubular morphology, pore volume, and enlarged interlayer spacing aid Li^+^ transport	[168]
Cyanobacterial biochar	K_2_CO_3_ modification	1.076 W m^−2^; 91.57% COD removal; 47.41% CE	Large active area and improved extracellular electron transfer	[167]
Corncob biochar	N doping/Co_3_O_4_ hybrid	ORR onset 0.84 V; limiting current −3.45 mA cm^−2^	Defect-assisted charge transfer and Co_3_O_4_ redox centers	[214]
g-C_3_N_4_/biochar	C incorporation and N-defect engineering	4.61 mmol g^−1^ h^−1^ H_2_O_2_	Charge separation and selective two-electron O_2_ reduction	[215]

**Table 4:** Quantitative comparison of representative environmental applications.

Feedstock/Material	Modification	Metric	Dominant Mechanism	Ref.
Fe–Mn biochar	Bimetal incorporation	96.7% atrazine removal	SO_4_•^−^/•OH plus non-radical pathways and Fe/Mn valence cycling	[238]
Lignocellulosic biochar	KOH activation	>2.35 mmol g^−1^ CO_2_ at 1 bar, 298 K	Micropore filling; governed by micropore volume, pore width, and polarity	[108]
Sugarcane leafy trash	MgO hybridization	157 mg g^−1^ As(V); 103 mg g^−1^ Pb(II); 297 mg g^−1^ MB	Precipitation/deposition, complexation, electrostatic and π interactions	[275]
Five feedstocks	300–600 °C pyrolysis	5.58–91.16 mg g^−1^ VOCs; 6.9–14.7% loss after 5 cycles	Partitioning at low T; pore filling at high T	[276]
Sugarcane bagasse	S-nZVI embedding	>90% dyes/microplastics within 15 min	Degradation, attraction, complexation, and π-π interactions	[298]
Willow catkins	Hydrophilic porous carbon membrane/tree-like configuration	1.65 kg m^−2^ h^−1^; 90.5% efficiency; 99.9% Na^+^ removal	Broadband absorption, capillary transport and heat localization	[299]

**Table 5:** Quantitative comparison of representative biochar-based sensors.

Feedstock/ Platform	Modification	Analyte and Metric	Performance Origin	Ref.
Biomass-derived carbon	CuO/g-C_3_N_4_ ternary composite	Malathion; LOD 1.2 pg mL^−1^; range 0.184–5.660 pg mL^−1^	Preconcentration, π interactions, CuO redox activity, and low charge-transfer resistance	[357,358]
Waste mushroom bran	N doping and activation–carbonation	Pb^2+^ LOD 0.0615 μM; Cd^2+^ LOD 0.118 μM	Higher area, N sites, functional groups, and conductivity	[358]

**Table 6:** Minimum comparison framework for scale-up and life-cycle assessment.

Comparison	Required Functional Unit	Critical Inventory Items	Decision Criterion
Biochar vs. nickel foam electrode	1 kWh delivered over full-cell life	Feedstock logistics, pyrolysis energy/yield, activation, deposited active material, mass loading, cycle life, recovery	Cost and impact per delivered energy, not per kilogram substrate
Biochar vs. commercial activated carbon	1 kg target removed at specified concentration	Precursor burden, heat, activator recovery, wash water, regeneration energy, capacity decay, end-of-life	Impact and cost per treatment service over all cycles
Conventional vs. plasma/microwave pyrolysis	1 kg on-spec biochar	Electricity mix, gas flow, residence time, throughput, yield, surface properties, reactor life	Energy, emissions, water, and cost at equivalent product quality

## Data Availability

All the data cited in this paper are derived from published literature and no new experimental data were generated.
